# Changes in norovirus genotype diversity in gastroenteritis outbreaks in Alberta, Canada: 2012–2018

**DOI:** 10.1186/s12879-019-3792-y

**Published:** 2019-02-19

**Authors:** Maria E. Hasing, Bonita E. Lee, Yuanyuan Qiu, Ming Xia, Kanti Pabbaraju, Anita Wong, Graham Tipples, Xi Jiang, Xiaoli L. Pang

**Affiliations:** 1 Department of Laboratory Medicine and Pathology, University of Alberta, Edmonton, AB Canada; 2 Department of Paediatrics, University of Alberta, Edmonton, AB Canada; 3 Division of Infectious Diseases, Cincinnati Children’s Hospital Medical Centre, Cincinnati, OH USA; 4 Department of Medical Microbiology and Immunology, University of Alberta, Edmonton, AB Canada; 5 Provincial Laboratory for Public Health (Microbiology), University of Alberta Hospital, WMC 2B4.58, 8440-112 Street, Edmonton, Alberta T6G 2J2 Canada

**Keywords:** Norovirus, Alberta, Canada, Gastroenteritis, Outbreaks, Humans, Molecular epidemiology

## Abstract

**Background:**

The emergence of norovirus genotype GII.4 variants has been associated with gastroenteritis pandemics worldwide, prompting molecular surveillance for early detection of novel strains. In this study, we aimed to analyze the outbreak activity of norovirus and characterize the norovirus strains circulating in Alberta between July 2012 and February 2018.

**Methods:**

Stool samples from gastroenteritis outbreaks in Alberta were tested for norovirus at the Provincial Laboratory for Public Health using a multiplex real time-RT PCR assay. The ORF1 and ORF2-genotypes of norovirus positive samples were assigned based on phylogenetic analyses of partial polymerase and capsid sequences, respectively.

**Results:**

A total of 530 norovirus outbreaks were identified. During July 2012 and June 2017 there was a gradual decrease in the annual number of GII.4 outbreaks, however, outbreak numbers increased from June 2017–February 2018. Four novel strains emerged: GII.17 Kawasaki in July 2014–June 2015, GII.P16/GII.4 Sydney in July 2015–June 2016, GII.P16/GII.2 and GII.P4 New Orleans/GII.4 Sydney in July 2016–June 2017. GII.Pe/GII.4 Sydney was the single predominant strain responsible for the majority (over 50%) of all norovirus outbreaks up to June 2015. Between June 2017 and February 2018, GII.P16/GII.4 Sydney was the leading strain causing 63% of all norovirus outbreaks.

**Conclusions:**

GII.4 stands as the predominant capsid genotype causing a large majority of the norovirus outbreaks in early 2018. An increase in genotype diversity was observed in the last years, characterized by a high circulation of non-GII.4 strains and GII.4 recombinants.

**Electronic supplementary material:**

The online version of this article (10.1186/s12879-019-3792-y) contains supplementary material, which is available to authorized users.

## Background

With advancement in molecular testing, Norovirus (NoV) is recognized as the leading cause of morbidity and mortality from diarrhoeal disease across all ages [1]. Approximately 18% of all acute gastroenteritis around the world is caused by NoV with an estimated economic burden of 60 billion US dollars per year [2, 3]. Norovirus has a single-stranded positive-sense RNA genome of ~ 7.5 Kb encompassing three open reading frames (ORFs). The three ORFs encode a non-structural polyprotein, the major capsid gene (VP1) and the minor capsid gene (VP2).

The NoV genome has high genetic variability at the P2 subdomain of VP1. P2 forms the outermost part of the virion and contains binding sites for histo-blood group antigens (HBGAs), attachment factors for human NoV strains. Norovirus can evade host immune responses by antigenic drift, through a process similar to influenza’s epochal evolution [4]. Recombination is another mechanism of evolution for NoV that often occurs at the ORF1/ORF2 junction, further increasing the genetic diversity of the virus [5]. Seven NoV genogroups (GI to GVII) have been described to date of which GI, GII and GIV can cause gastroenteritis in humans, with GIV being less common [6]. NoV genogroups are sub classified into genotypes; at least 14 ORF1-based and 9 ORF2-based genotypes have been described for GI and 27 ORF1-based and 22 ORF2-based genotypes for GII [6].

NoV GII.4 has been the most common genotype in circulation worldwide since the mid-1990s. New genetic clusters or variants of NoV GII.4 have emerged periodically and caused pandemics including: US95/96 in 1996, Farmington Hills in 2002, Hunter in 2004, Den Haag in 2006, New Orleans in 2010 and Sydney in 2012 [7–9]. No global pandemic GII.4 strain has yet emerged after GII.4 Sydney. A novel cluster of GII.17 named Kawasaki became predominant in Asia during the 2014–2015 winter season [10] raising concern over a possible new global pandemic NoV genotype. Since its emergence in Asia, GII.17 Kawasaki has also been reported in other continents although with lower prevalence [10].

It is yet unknown why GII.4 has remained as the predominant genotype for over two decades but several features of this genotype support its enhanced circulation, including higher rates of evolution and a progressive accumulation of mutations that help evade host immune responses [11, 12]. Moreover, pandemic GII.4 variants can broadly bind to a wide set of HBGAs, a feature that favours virus transmission by providing NoV a large pool of individuals genetically susceptible to infection [13].

The aim of the present study is to describe the outbreak activity of NoV from July 2012 to February 2018 in Alberta, Canada. We provide information on the norovirus genotypes in circulation and their relevance in outbreak settings. This study provides important data for vaccine development and enhances our understanding of norovirus disease burden.

## Methods

### Samples

Gastroenteritis outbreak investigations in Alberta were managed by public health officials in collaboration with the Provincial Laboratory for Public Health (ProvLab) [8]. Stool samples collected between July 2012 and February 2018 during outbreak investigations were tested at ProvLab for NoV genogroup I and genogroup II using a real time RT-PCR assay [14]. A NoV- confirmed outbreak was defined as ≥2 epidemiologically linked cases with gastroenteritis and at least one sample tested positive for NoV. Data was analyzed from July to June of the following year considering the winter seasonality of NoV.

Outbreak settings were classified into 6 different groups: 1) community long-term care, hospital long-term care, supportive living, and group homes facilities; 2) hospital acute care; 3) food establishments, catering events, food shops, community functions, conferences and hotels; 4) day care centers; 5) other types of group residences, e.g. camp, dormitory; and 6) other settings including community shelters, community services, household, schools and cruise ships. Groups 1 and 2 were classified as healthcare-related settings, whereas groups 3 to 6 were classified as non-healthcare-related.

### Norovirus genotyping

For each NoV-positive outbreak, one NoV positive sample was selected for genotyping. Briefly, the nucleic acid extract from each stool sample was subjected to reverse transcription (RT) with random primers and the resulting cDNA was PCR-amplified. Samples collected up to February 2017, were amplified in region C (ORF2) using primer pair G2SKF/G2SKR for genogroup II strains or primer pair G1SKF/G1SKR for genogroup I strains [15]. Samples with emergent or unassigned genotypes based on region C sequence analysis were further genotyped targeting the 3’end of the polymerase gene using primers LV4282-99F [16] and COG2R [17]. Samples collected from March 2017 onwards, were genotyped using a dual polymerase-capsid genotyping protocol based on a single PCR amplicon obtained with primer pair MON432/G1SKR for genogroup I strains and primer pair MON431/G2SKR for genogroup II strains [18]. All PCR products were subjected to Sanger sequencing and genotypes were assigned using the Norovirus Genotyping tool [19]. A large majority of strains from outbreaks occurring between July 2012 and June 2015 were left uncharacterized at ORF1; retrospective characterization was not attempted based on observations that 69% of norovirus GII outbreaks in Alberta were caused by a single ORF2 genotype, GII.4 Sydney, and reports from North America [18] and diverse countries from different continents [20] suggest that GII.Pe/GII.4 Sydney was the major strain in circulation worldwide during that time frame.

### Phylogenetic analysis

Phylogenetic analyses of GII.17, GII.P16/GII.4 Sydney, GII.P4 New Orleans/GII.4 Sydney and GII.P16/GII.2 sequences obtained in our study were performed with MEGA 6.06 [21]. Maximum likelihood trees were constructed using the substitution model producing the lowest Bayesian Information Criterion scores, as calculated by the software. For all trees, branch significance was estimated based on 1000 bootstrap replicates.

### Cloning, expression and purification of recombinant P domain proteins

The capsid P domain of two GII.P16/GII.4 Sydney outbreak strains (AB-2016-26 and AB-2016-190) were amplified by RT-PCR using forward primer ACGCGGATCCTCAAGAACTAAACCATTCTCTGTCC and reverse primer ATAAGAATGCGGCCGCTTAGCAAAAGCAATCGCCACGGCAATCGCATACTGCACGTCTACGCCCCGTTCC and cloned into pGEX-4 T-1 vector (GST Gene fusion System, GE Healthcare Life Sciences) between the Bam HI and Not I sites. An RGD4C tag (CDCRGDCFC) was linked to the C terminus of the P domain for P particle formation [22]. The recombinant P domain protein was expressed in *E. coli* (BL21, DE3) with induction by 0.25 mM isopropyl-β-D-thiogalactopyranoside (IPTG) at room temperature (~ 21 °C) overnight as described elsewhere [22]. Purification of the glutathione S-transferase (GST)-P domain fusion protein was performed using resin of Glutathione Sepharose 4 Fast Flow (GE Healthcare Life Sciences) according to the manufacturer’s instruction. GST was removed from the target proteins by thrombin (GE Healthcare Life Sciences) cleavage either on beads or in solution (phosphate buffer saline, PBS, pH 7.4).

### Saliva binding assay of P-domain proteins

The saliva-based binding assays were performed as previously described [13]. Briefly, boiled human saliva with known HBGA phenotypes collected from Cincinnati, OH, United States, were diluted 1000-fold and used to coat 96-well microtiter plates (Dynex Immulon; Dynatech, Franklin, MA). After blocking with 5% non-fat milk in PBS, different concentrations of P-domain protein (15, 7.5, 3.75 ng/μl) were added to the wells. The bound P proteins were detected using a guinea pig anti-NoV antiserum (1:3000), followed by the addition of HRP-conjugated goat anti-guinea pig IgG. The HRP activity was then measured with TMB kit (Kierkegaard & Perry Laboratories, Gaithersburg, MD) and the OD450 values were read with an ELISA spectrum reader (Tecan, Durham, NC).

### Statistical analysis

The proportion of NoV GI and GII outbreaks by settings were compared using the Chi-square exact test. The annual numbers of NoV positive outbreaks occurring between July 2012 and June 2017 were compared to those occurring in the previous 5 years, from July 2007 to June 2012, using a one tailed t-test, and a significance of *p* < 0.05.

## Results

### Norovirus outbreaks: Annual activity and circulating genotypes

**Fig. 1:**
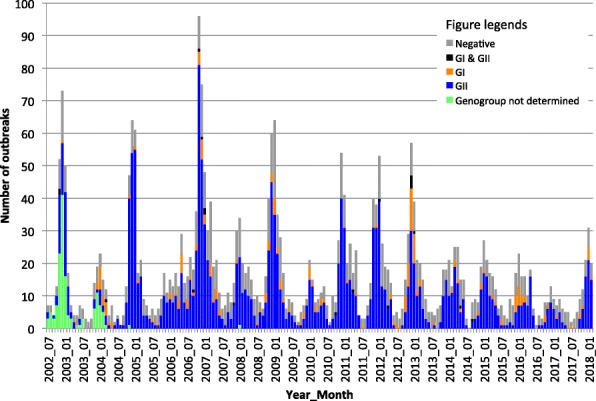
Monthly distribution of norovirus-positive outbreaks in Alberta by genogroup. Data from years July 2002 to June 2012 were reported previously by Pang et al. [31] and Hasing et al. [8]. The data from this study corresponds to the period July 2012 to February 2018

A total of 1572 gastroenteritis outbreak investigations were performed in Alberta between July 1st 2012 and February 30th 2018, of which 859 (54.6%) had specimens submitted to the ProvLab for laboratory testing. Norovirus was identified in 530 (61.7%) of all tested outbreaks. The monthly distribution of NoV-positive outbreaks peaked in the winter months (Fig. 1), however, spring peaks with higher activity than that of winter months occurred in March 2014 and May 2016. Compared to historical data, the annual numbers of NoV outbreaks between July 2012 to June 2017 were lower than those observed in the previous 5 years (July 2007 to June 2012 vs. July 2012 to June 2017, *p* = 0.0489, one-tailed t-test).

**Fig. 2:**
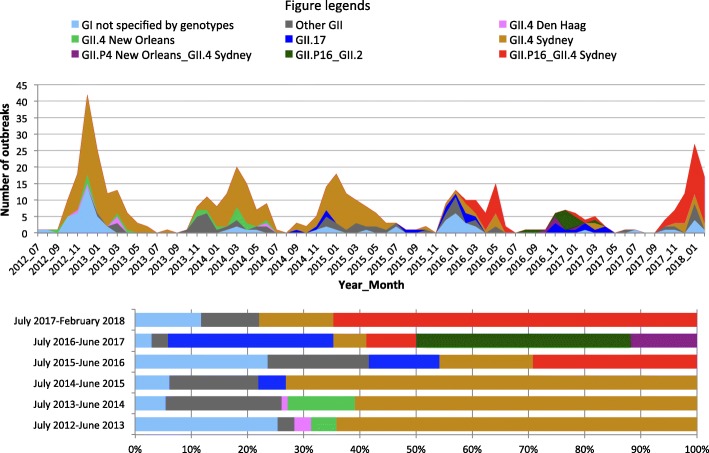
Monthly and annual distribution of norovirus outbreaks in Alberta by genotypes

Genogroup II strains were responsible for 440 out of 530 (83.0%) of laboratory confirmed NoV outbreaks (Table 1), while genogroup I and mixed genogroup I and genogroup II strains were responsible for 83 (15.7%) and 7 (1.3%) NoV outbreaks, respectively. Twenty ORF2-based genotypes were identified throughout the study period: GI.1, GI.2, GI.3, GI.4, GI.5, GI.6, GI.7, GI.9, GII.1, GII.2, GII.3, GII.4, GII.5, GII.6, GII.7, GII.8, GII.13, GII.14, GII.16 and GII.17 (Table 2). Overall, GII.4 was the most common genotype and was responsible for at least 319 (60.2%) out of 530 NoV-positive outbreaks. Strains carrying an ORF2 of variant Sydney were accountable for the majority of GII.4 outbreaks (297/319, 93.1%). GII.4 variants Den Haag and New Orleans, which caused pandemics in 2006 and 2009, respectively, disappeared after June 2014. Genotypes GI.6 and GI.7 had an increased presence during July 2012 to June 2013 whereas GI.3 was the predominant GI strain from July 2015 to June 2016. Four novel NoV strains emerged during the last three annual periods of study (Fig. 2): 1) GII.17; 2) GII.P16/GII.4 Sydney, a recombinant strain carrying a polymerase gene of GII.16 and a GII.4 Sydney capsid gene; 3) GII.P16/GII.2, a recombinant strain carrying a GII.16 polymerase gene and a GII.2 capsid gene and 4) GII.P4 New Orleans/GII.4 Sydney, a recombinant strain with a GII.4 New Orleans polymerase gene and a GII.4 Sydney capsid gene.

**Table 1:** Distribution of norovirus outbreaks by genogroups

Genogroup	July 2012–June 2013	July 2013–June 2014	July 2014–June 2015	July 2015–June 2016	July 2016–June 2017	July 2017- Feb 2018	Total
GI	40	6	6	18	4	9	83
GII	103	96	85	60	35	61	440
Mixed GI and GII	5	1	1	0	0	0	7
TOTAL	148	103	92	78	39	70	530
GI sequenced (%)	34 (75.6)	5 (71.4)	5 (71.4)	17 (94.4)	1 (25)	8 (88.9)	70 (77.8)
GII sequenced (%)	100 (92.6)	87 (89.7)	77 (89.5)	55 (91.7)	33 (94.3)	60 (98.4)	412 (92.2)

**Table 2:** Distribution of Norovirus strains by ORF1 and ORF2 genotype combinations

ORF1/ORF2 genotypes	July 2012–June 2013	July 2013–June 2014	July 2014–June 2015	July 2015–June 2016	July 2016–June 2017	July 2017- Feb 2018	Total
GI sequenced							
Pol NT/GI.1			1				1
GI.P1/GI.1						3	3
Pol NT/GI.2		2		1			3
GI.2/GI.P2					1		1
Pol NT/GI.3		1	1	15			17
GI.P3/GI.3						4	4
Pol NT/GI.4		1					1
Pol NT/GI.5			2				2
Pol NT/GI.6	20		1				21
GI.Pb/GI.6						1	1
Pol NT/GI.7	14			1			15
Pol NT/GI.9		1					1
GII sequenced							
Pol NT/GII.1				1			1
GII.P16/GII.2					13		13
GII.P2/GII.2	1			1			2
GII.Pe/GII.2				1			1
Pol NT/GII.3				1			1
GII.P12/GII.3					1	3	4
Pol NT/GII.4 Den Haag 2006b	4	1					5
Pol NT/GII.4 New Orleans 2009	5	7					12
GII.P4 New Orleans 2009/GII.4 New Orleans 2009	1	4					5
Pol NT/GII.4 Sydney 2012	86	54	51	9		4	204
GII.P16/GII.4 Sydney 2012				21	3	44	68
GII.P4 New Orleans 2009/GII.4 Sydney 2012					4		4
GII.Pe/GII.4 Sydney 2012		2	9	3	2	5	21
Pol NT/GII.5		7	1				8
Pol NT/GII.6	2	7	5	5			19
GII.P7/GII.6						3	3
Pol NT/GII.7	1		1	2			4
GII.P7/GII.7						1	1
Pol NT/GII.8		1	1	1			3
Pol NT/GII.13		4	4				8
Pol NT/GII.14			1				1
Pol NT/GII.16				1			1
Pol NT/GII.17			4	9	6		19
GII.P17/GII.17					4		4

Pol NT = polymerase not typed

### NoV GII.17 outbreaks in Alberta

The first GII.17 outbreak in Alberta occurred in September 2014. Phylogenetic analysis of complete capsid sequences identified all GII.17 strains as part of the Kawasaki cluster (Additional file 1: Figure S1). Representatives of two sub-clusters of GII.17 Kawasaki previously defined by Chan et al. [23] were identified: strains from 2014 grouped into the Kawasaki323-like sub-cluster (0.7–1% nucleotide and 0.7–0.9% amino acid p-distances with AB983218), and strains from 2015 and 2016 grouped into the Kawasaki308-like sub-cluster (0.4–0.9% nucleotide and 0.2–0.6% amino acid p-distances with LC037415). GII.17 was responsible for 4.3, 11.5 and 25.6 (%) of all NoV outbreaks during the periods of July 2014–June 2015, July 2015–June 2016 and July 2016–June 2017, respectively. No GII.17 outbreaks were observed after April 2017.

### Novel GII.P16/GII.4 Sydney recombinant

**Fig. 3:**
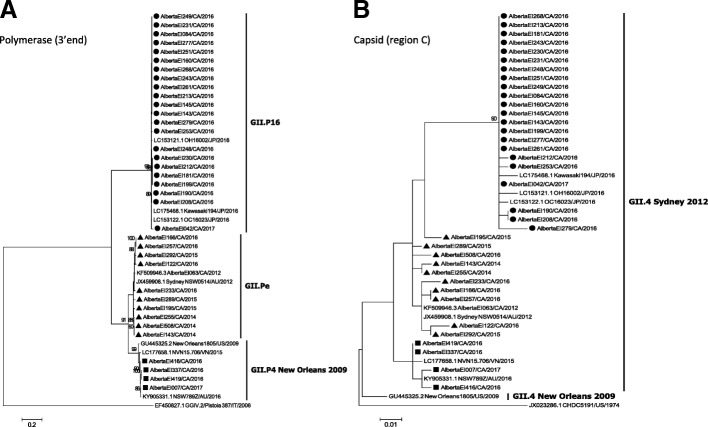
Maximum likelihood phylogenies of recombinant GII.4 strains that emerged in Alberta. Three different GII.4 Sydney recombinant strains were identified based on sequences of (**a**) the 3’end of ORF1 and (**b**) region C of the capsid gene. The novel GII.P16/GII.4 Sydney strains are shown with solid circles; GII.P4 New Orleans/GII.4 Sydney and GII.Pe/GII.4 Sydney strains are shown with solid squares and solid triangles, respectively. Trees A and B were constructed using the Kimura-2 parameter substitution model assuming gamma-distributed rates of evolution among sites with (tree A) and without (tree B) invariant sites. Branch significance was estimated based on 1000 bootstrap replicates

Several GII.4 strains causing outbreaks in early 2016 could not be classified at the variant level by the genotyping tool based solely on region C sequences. BLAST analysis with additional sequence data from the 3’end of ORF1 (~ 740 nt) revealed a high nucleotide identity (99%) with Kawasaki194-like sequences (GenBank accession number LC175468), previously characterized as GII.P16/GII.4 Sydney recombinant strains [24]. Phylogenetic trees of partial ORF1 and partial ORF2 sequences of this recombinant are shown on Fig. 3. The complete capsid sequences of four GII.P16/GII.4 Sydney recombinants collected between 2016 and 2018, AlbertaEI84/2016, AlbertaEI277/2016, AlbertaEI487/2017 and AlbertaEI231/2018, had 3.3–3.9% nucleotide and 1.5–2.2% amino acid p-distances with the GII.Pe/GII.4 Sydney 2012 reference strain NSW0514/AU/2012 (accession number JX459908) (Additional file 2: Figure S2). Compared with GII.Pe/GII.4 Sydney, the GII.P16/GII.4 Sydney strains did not present distinctive mutations at the P2 domain, however, all GII.P16/GII.4 Sydney sequences in our data set shared two isoleucines at residues 119 and 145, both located at the S domain (Additional file 3: Figure S3). The first GII.P16/GII.4 Sydney outbreak in Alberta occurred in February 2016. Overall, the GII.P16/GII.4 Sydney recombinant was responsible for 21 (27.0%) of 78 NoV outbreaks from July 2015–June 2016, 3 (7.7%) out of 39 NoV outbreaks from July 2016–June 2017 and 44 (62.9%) out of 70 NoV outbreaks from July 2017–February 2018.

### Saliva binding profile of the novel GII.P16/GII.4 Sydney recombinant strain

In order to investigate the HBGA binding properties of the novel GII.P16/GII.4 Sydney recombinant, we performed binding assays using p-particles of two representative strains, AB-2016-26 and AB-2016-190 and saliva of individuals with known HBGA phenotypes. Both strains demonstrated ability to bind saliva of A, B and O secretors and did not bind saliva of non-secretors (Additional file 4: Figure S4). AB-2016-190 showed stronger binding to secretors than AB-2016-26, but both strains showed no binding to non-secretors, similar to the binding pattern of Syd9-2B, a GII.Pe/GII.4 Sydney strain.

### Novel GII.P16/GII.2 recombinant

**Fig. 4:**
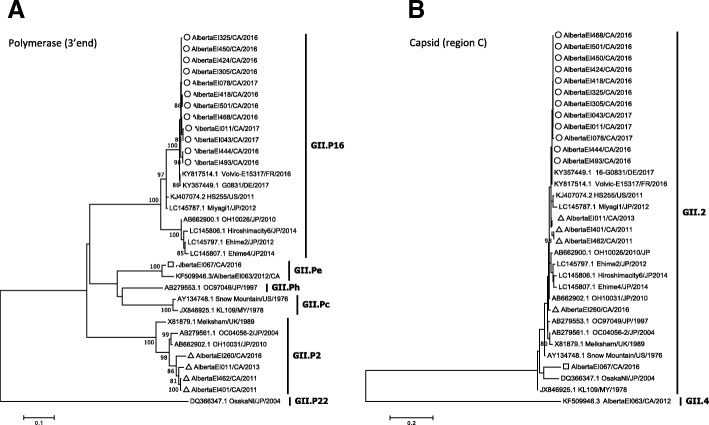
Maximum likelihood phylogenies of norovirus GII.2 strains. GII.2 sequences at (**a**) the 3’end of ORF1 and (**b**) region C of the capsid gene were analyzed. The novel GII.P16/GII.2 recombinant sequences are shown with open circles; GII.Pe.GII.2 and GII.P2/GII.2 strains are shown, respectively, with open squares and open triangles. All trees were constructed using the Kimura-2 parameter substitution model assuming gamma-distributed rates of evolution among sites with (tree A) and without (tree B) invariant sites. Branch significance was estimated based on 1000 bootstrap replicates

Based on retrospective ORF1-genotyping analysis of GII.2 outbreaks back to November 2011, we identified that the first GII.P16/GII.2 outbreak in Alberta occurred in August 2016. In BLAST analysis, the GII.P16/GII.2 outbreak strain shared highest identity (99%) with Volvic-E15317, a strain collected in France in late 2016 (Fig. 4). GII.P16/GII.2 was the predominant NoV strain between July 2016–June 2017 (13 of 39 NoV outbreaks, 33.3%).

### GII.P4 New Orleans/GII.4 Sydney recombinant

The recombinant strain GII.P4 New Orleans/GII.4 Sydney was identified during the period July 2016–June 2017 (Fig. 3). The strain was responsible for four out of five GII.4 outbreaks from that period and was not observed after June 2017.

### NoV outbreak settings

Between July 2012 and February 2018, the majority of outbreaks occurred in community long-term care, hospital long-term care, supportive living, and group homes facilities (63/83, 75.9% of all NoV GI and 346/440, 78.6% of all NoV GII outbreaks, respectively) and hospital acute care (5/83, 6% of GI and 53/440,12% of GII outbreaks) (Additional file 5: Figure S5). A larger proportion of GI outbreaks occurred in non-health care settings compared to GII (15/83, 18.1% vs. 41/440, 9.3%, *p* = 0.0180, Chi-square test).

## Discussion

During July 2012 to February 2018 the NoV outbreak activity in Alberta presented important changes in terms of overall disease burden and NoV genotype distribution as compared to historical data. Between July 2012 and June 2017, the annual numbers of NoV outbreaks had a gradual decline. Genotype GII.4 outbreaks, which have previously represented over 50% of all NoV outbreaks, decreased to 23% in July 2016–June 2017. The steep decline in NoV outbreaks was followed by rebound in July 2017–February 2018, when GII.4 outbreaks were accountable for 76% of all NoV outbreaks.

GII.4 Sydney remained as the single predominant strain responsible for a large majority (> 50%) of all NoV outbreaks from July 2012 to June 2015. In contrast, the following 2 years, July 2015 to June 2017, had an atypical high proportion of non-GII.4 outbreaks. Moreover, four novel NoV strains emerged in the province: GII.17 Kawasaki, GII.P16/GII.4 Sydney, GII.P16/GII.2, and GII.P4 New Orleans/GII.4 Sydney.

The GII.17 Kawasaki strain was first reported in Asia in September 2014 where it quickly became the predominant genotype during the 2014–2015-winter season raising concern of a global pandemic [10]. Since its emergence, GII.17 Kawasaki has undergone diversification into at least three sub-clusters [10]. In Alberta, we identified two sub-clusters: the Kawasaki 308-like sub-cluster and the Kawasaki 323-like sub-cluster. Although both clusters started to circulate in the province at similar timeframes as reported in Asia [10], the GII.17 Kawasaki had limited prevalence in Alberta, as also observed in other regions outside Asia, including Europe and the United States [10]. The factors limiting the transmission of GII.17 Kawasaki outside Asia are largely unknown. The GII.17 Kawasaki strains display a broad saliva HBGA-binding profile comparable to pandemic GII.4 strains [25], which emphasizes the potential of GII.17 as a pandemic strain.

The novel GII.P16/GII.4 Sydney recombinant was the most common strain among NoV outbreaks from July 2015–June 2016. Although the prevalence of this recombinant decreased temporarily in July 2016–June 2017, its circulation increased sharply in the more recent period, July 2017 and February 2018, when GII.P16/GII.4 Sydney was again responsible for a large majority of outbreaks. The GII.P16/GII.4 Sydney recombinant also circulated in the United States and Germany during the 2015–2016 winter season [18, 26]. Our analysis of capsid sequences from this recombinant collected at three different time points (2016, 2017 and 2018) demonstrated no unique amino acid substitutions compared to GII.Pe/GII.4 Sydney, its ORF2 parental strain. Furthermore, we observed that GII.P16/GII.4 Sydney can bind to saliva of secretor HBGA phenotypes but not to that of non-secretors, similarly as GII.Pe/GII.4 Sydney strains. Our findings agree with a recent phylogenetic study [27] and suggest that amino acid substitutions outside ORF2, rather than antigenic change at the capsid gene, conferred an evolutionary advantage to GII.P16/GII.4 Sydney over its parental strain GII.Pe/GII.4 Sydney.

The GII.P4 New Orleans/GII.4 Sydney recombinant had low circulation between July 2016 and June 2017 and was no longer observed in the subsequent period. We hypothesize that the transmission of this recombinant in Alberta might have been restricted by its antigenic resemblance to two highly circulated strains, GII.4 New Orleans and GII.4 Sydney. GII.4 has been the predominant genotype worldwide among humans since the 1990s but had very limited circulation in the 1970s and 1980s [28, 29]. It is possible that the decline in GII.4 NoV outbreaks observed between July 2015 and June 2017 is a consequence of herd immunity accumulated in the population after two decades of high prevalence of various GII.4 variants and exhaustion of mutational sites in GII.4 Sydney. The GII.P16/GII.4 Sydney recombinant might have partially circumvented such factors by acquiring the ORF1 genes from the less circulating, and presumably more fit, GII.P16. Further surveillance data is still required to understand the turnover of NoV genotypes in humans.

The last recombinant that emerged in Alberta in the time period of the present study is GII.P16/GII.2. Although GII.P16/GII.2 outbreaks have been reported previously in Asia in 2009, phylogenetic time-scale analyses performed by others suggest that the polymerase of the recent GII.P16/GII.2 strain is rather closely related to that of GII.P16/GII.4 Sydney and carries amino acid substitutions that could have conferred the novel GII.P16/GII.2 an evolutionary advantage [30]. GII.P16/GII.2 strains have also been reported in Germany, France, Japan, China and the United States since mid-2016 [18, 30]. Notably, GII.P16/GII.2 was the predominant strain in the province briefly in July 2016–June 2017, but was replaced thereafter by GII.P16/GII.4 Sydney.

A limitation of our study is the limited collection of ORF1 data for the period between July 2012 and June 2015, which could have lead us to miss novel or unusual recombinants. However, we believe the likelihood of missing important strains is low since 69% of the norovirus GII outbreaks in Alberta within those years were caused by a single ORF2 genotype, GII.4 Sydney. Reports from North America [18] and diverse countries around different continents [20] suggest that GII.Pe/GII.4 Sydney was the major strain in circulation worldwide during that time frame and thus, we believe that most, if not all of GII.4 Sydney from that period, carried a GII.Pe polymerase.

## Conclusions

In summary, we provided 6 years of systematic molecular surveillance data of norovirus outbreaks in Alberta and identified a GII.4 recombinant, GII.P16/GII.4 Sydney, as the most prevalent strain causing outbreaks in the province in early 2018. An important shift and increase in genotype diversity was noticed in recent years, which should be considered for vaccine development. Ongoing surveillance of the molecular epidemiology of NoV using a dual ORF1/ORF2 genotyping scheme is indispensable to better identify the disease burden of new emerging strains and understand their evolutionary pathways.

## Additional files

Additional file 1: **Figure S1.** Maximum likelihood phylogeny of GII.17 strains based on complete ORF2 sequences. GII.17 strains from outbreaks in Alberta are shown with triangles. A) Tree based on nucleotide sequences constructed using the Tamura-Nei substitution model assuming gamma-distributed rates of evolution among sites B) Tree based on amino acid sequences constructed using the Jones-Taylor-Thornton (JTT) substitution model assuming gamma-distributed rates of evolution among sites. Both trees were rooted using a GII.13 outgroup reference sequence. Branch significance was estimated based on 1000 bootstrap replicates. Clusters and subclusters are shown as defined in previous studies [31, 32] (PDF 92 kb)

Additional file 2: **Figure S2.** Maximum likelihood phylogeny of complete GII.P16/GII.4 Sydney capsid sequences. The maximum-likelihood tree was constructed using the Tamura-Nei substitution model, assuming gamma-distributed rates of evolution among sites. The analysis included strains representative of different countries and different years. (PDF 14 kb)

Additional file 3: **Figure S3.** Description of data: Analysis of variable sites in capsid sequences of GII.P16/GII.4 Sydney recombinants compared to GII.Pe/GII.4 Sydney strains. Tables depict all variable amino acid residues identified in the alignment used to construct the tree shown in Additional file 2. Evolving amino acid positions previously reported by Lindesmith et al. [33] to play a role in the evasion of antibody immune responses are shown in red. (PDF 80 kb)

Additional file 4: **Figure S4.** Saliva-binding of NoV P-domain proteins. The P-domain proteins of (A) AB-2016-26 (GII.P16/GII.4 Sydney), (B) AB-2016-190 (GII.P16/GII.4 Sydney) and (C) Syd9-2B (GII.Pe/GII.4 Sydney) were tested in their ability to bind saliva from individuals with different HBGAs profiles. (PDF 255 kb)

Additional file 5: **Figure S5.** Norovirus outbreak settings in Alberta by genogroup. Outbreaks from mixed GI and GII strains (*n* = 7) were excluded from the analysis. (PDF 23 kb)

## Abbreviations

GST: Glutathione S-transferase
HBGAs: Histo-blood group antigens
HRP: Horseradish peroxidase
NoV: Norovirus
ORF: Open reading frame
ProvLab: Provincial Laboratory for Public Health
RT: Reverse transcription
