# Supplementary figures and images for: Changes in norovirus genotype diversity in gastroenteritis outbreaks in Alberta, Canada: 2012–2018
Source: BMC Infect Dis. 2019 Feb 19;19:177. doi: 10.1186/s12879-019-3792-y (PMC6381812; doi:10.1186/s12879-019-3792-y)

A.

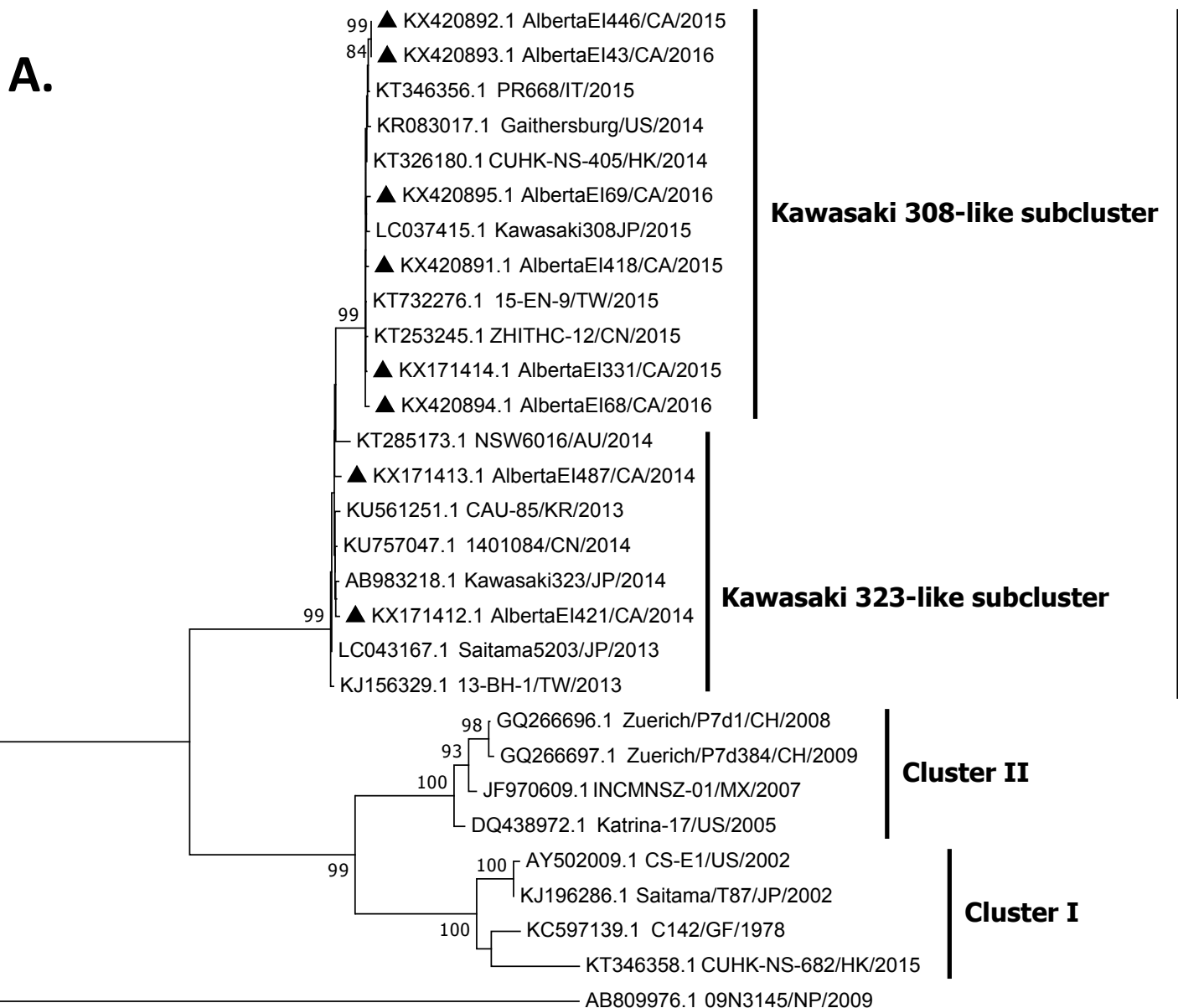

B.

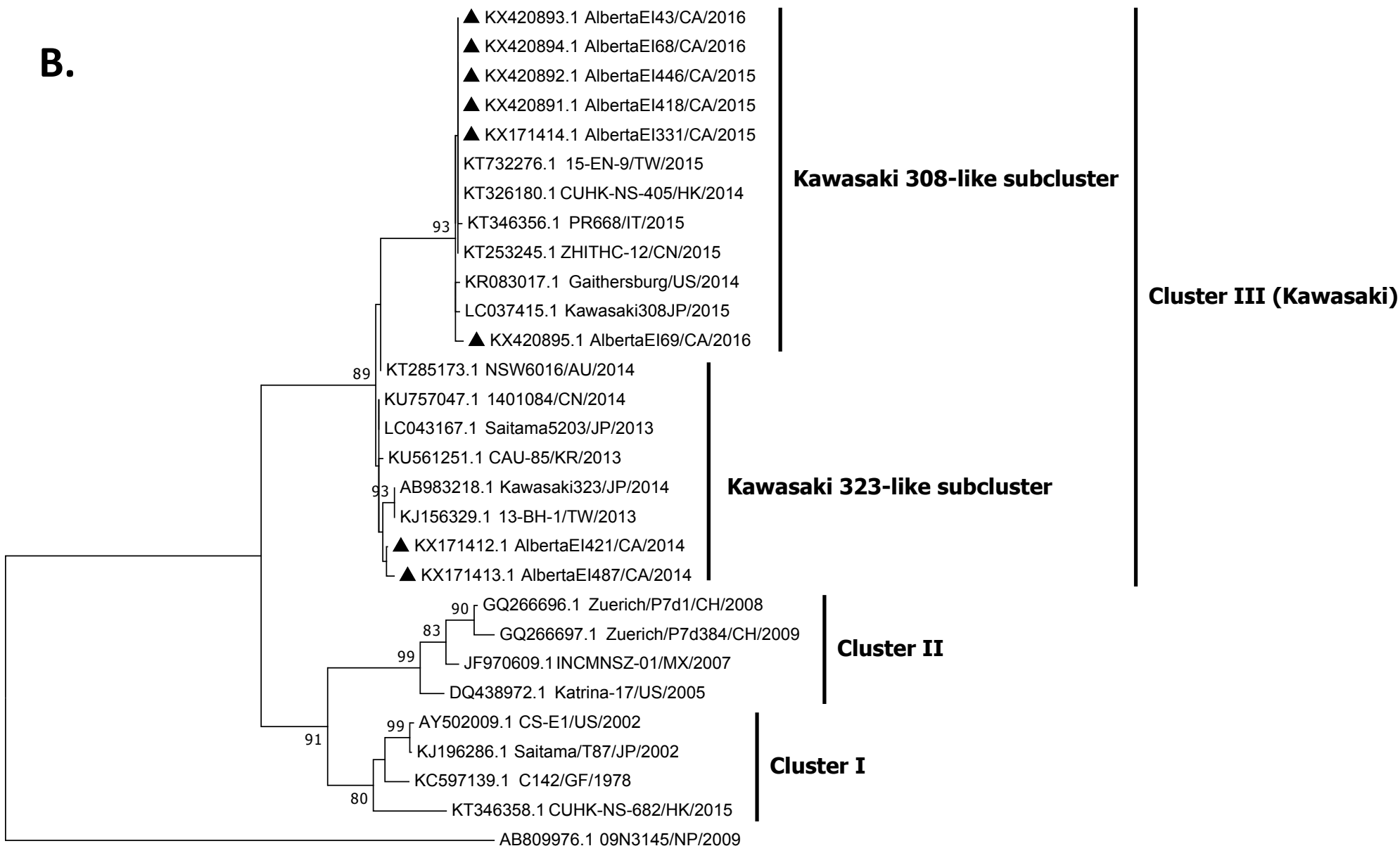

Supplement: Supplementary file 1 — Figure S1. Maximum likelihood phylogeny of GII.17 strains based on complete ORF2 sequences. GII.17 strains from outbreaks in Alberta are shown with triangles. A) Tree based on nucleotide sequences constructed using the Tamura-Nei substitution model assuming gamma-distributed rates of evolution among sites B) Tree based on amino acid sequences constructed using the Jones-Taylor-Thornton (JTT) substitution model assuming gamma-distributed rates of evolution among sites. Both trees were rooted using a GII.13 outgroup reference sequence. Branch significance was estimated based on 1000 bootstrap replicates. Clusters and subclusters are shown as defined in previous studies [31, 32] (PDF 92 kb) [file 12879_2019_3792_MOESM1_ESM.pdf]

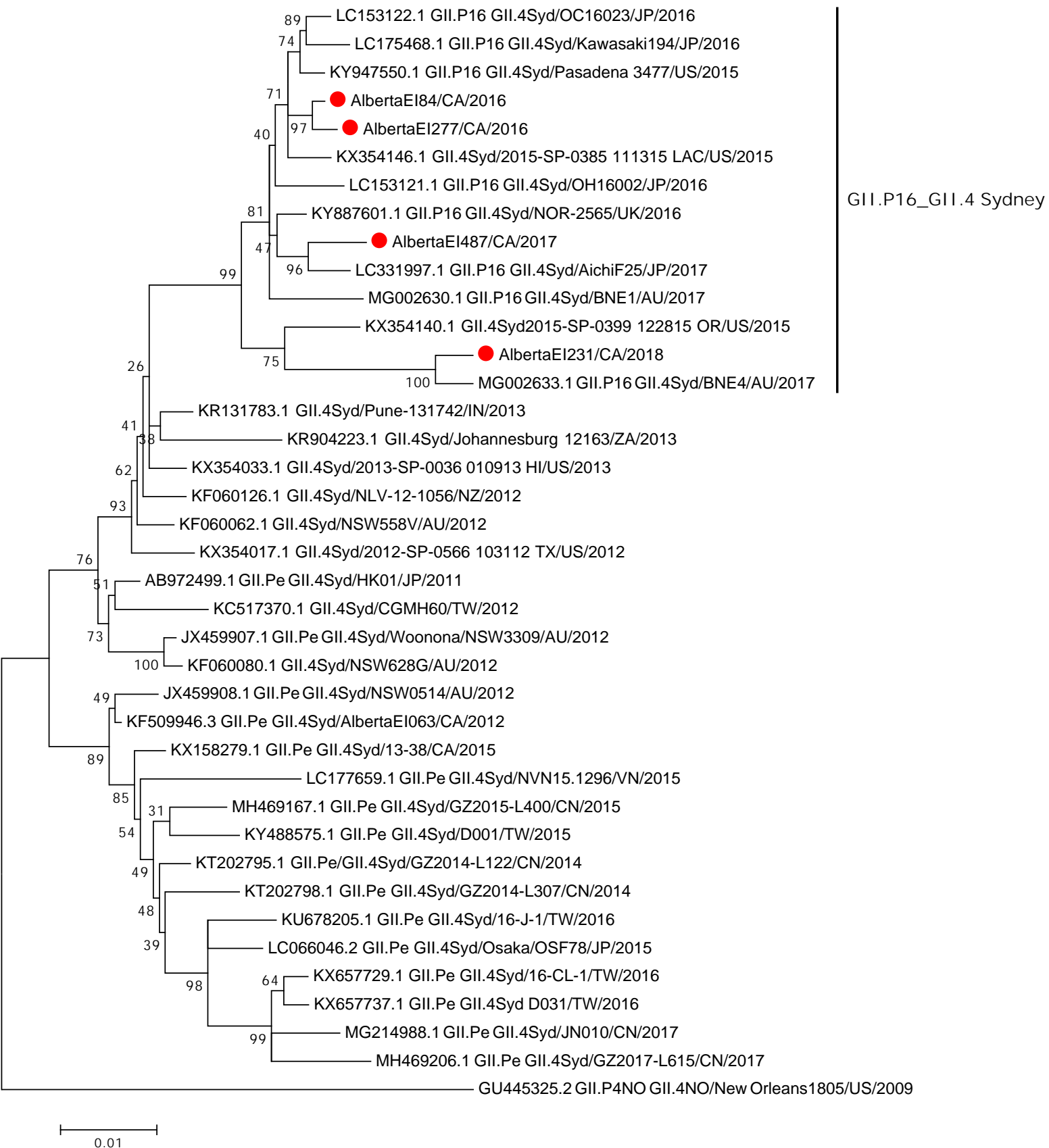

Supplement: Supplementary file 2 — Figure S2. Maximum likelihood phylogeny of complete GII.P16/GII.4 Sydney capsid sequences. The maximum-likelihood tree was constructed using the Tamura-Nei substitution model, assuming gamma-distributed rates of evolution among sites. The analysis included strains representative of different countries and different years. (PDF 14 kb) [file 12879_2019_3792_MOESM2_ESM.pdf]

A.

AB-2016-26

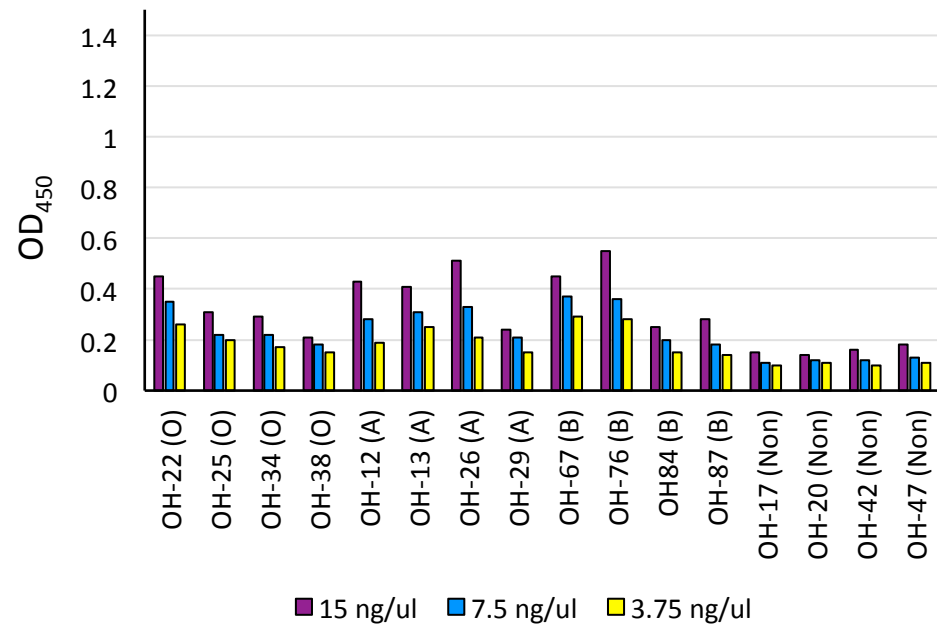

B.

AB-2016-190

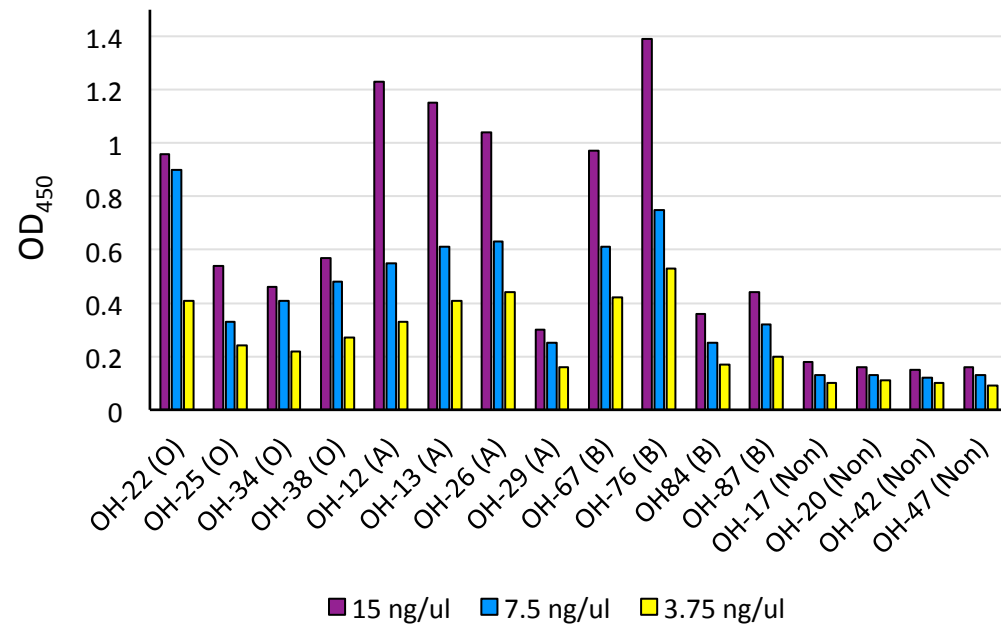

C.

Syd9-2B

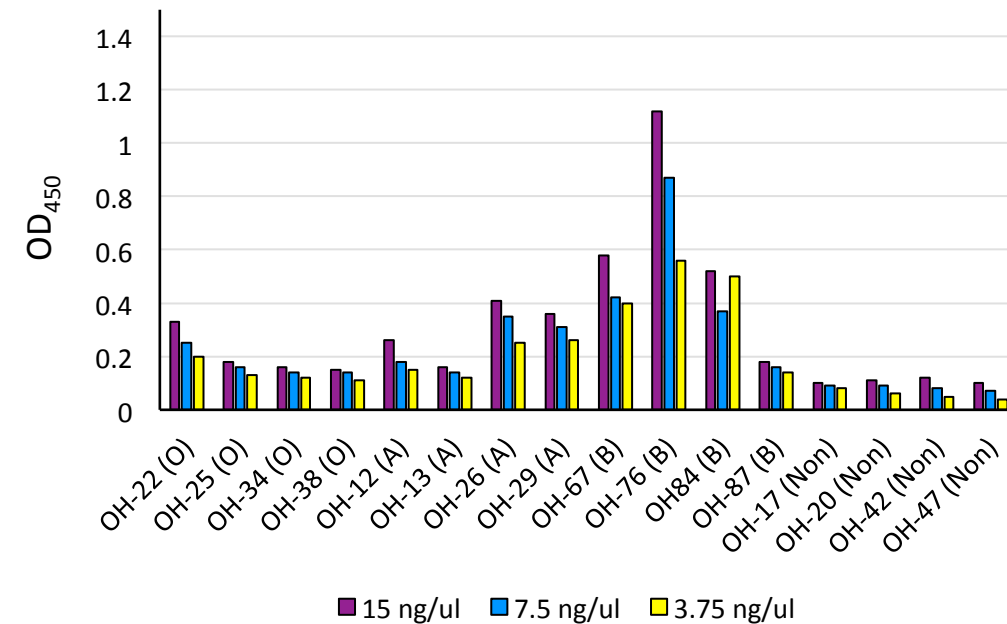

Supplement: Supplementary file 4 — Figure S4. Saliva-binding of NoV P-domain proteins. The P-domain proteins of (A) AB-2016-26 (GII.P16/GII.4 Sydney), (B) AB-2016-190 (GII.P16/GII.4 Sydney) and (C) Syd9-2B (GII.Pe/GII.4 Sydney) were tested in their ability to bind saliva from individuals with different HBGAs profiles. (PDF 255 kb) [file 12879_2019_3792_MOESM4_ESM.pdf]

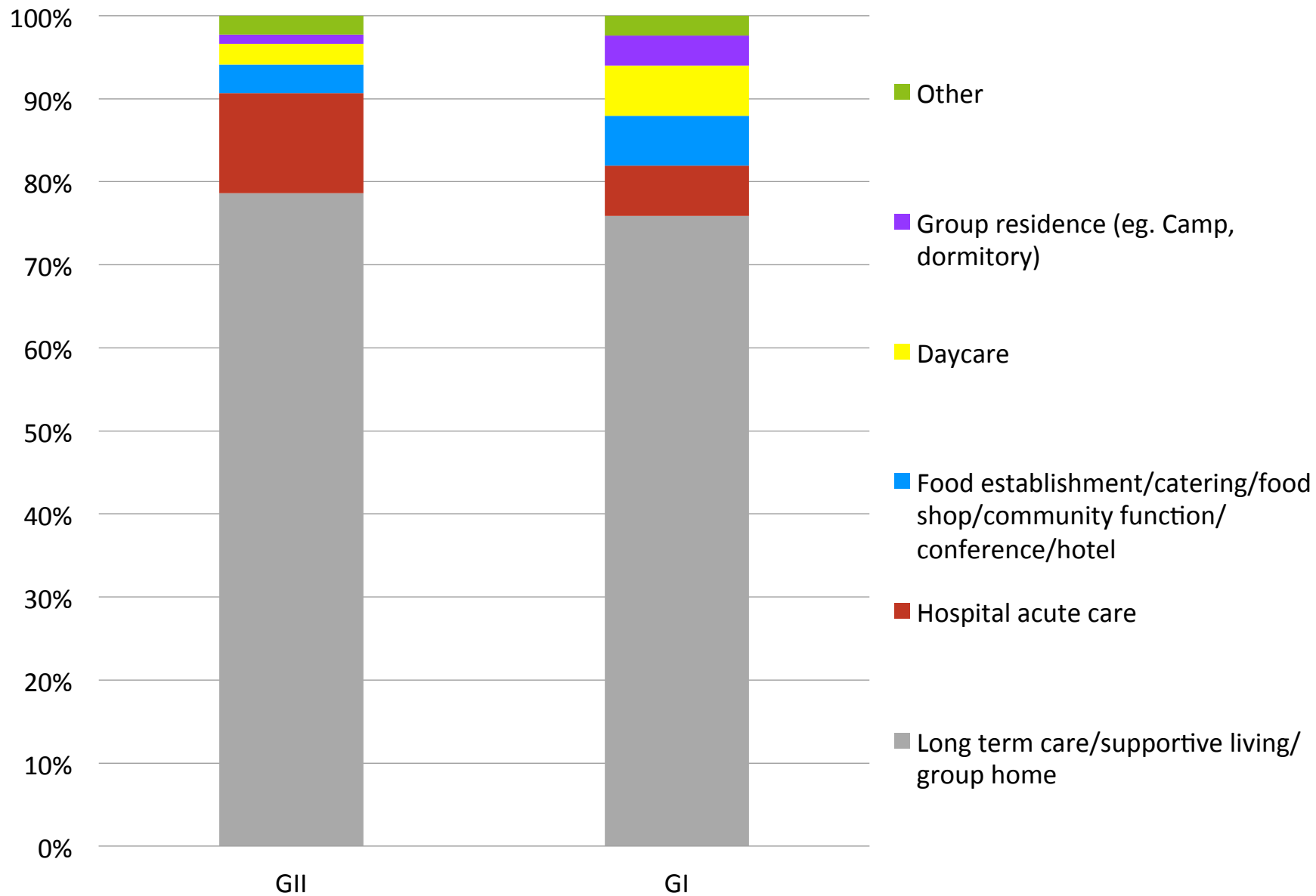

Supplement: Supplementary file 5 — Figure S5. Norovirus outbreak settings in Alberta by genogroup. Outbreaks from mixed GI and GII strains (n = 7) were excluded from the analysis. (PDF 23 kb) [file 12879_2019_3792_MOESM5_ESM.pdf]
