# Supplementary material for: Changes in norovirus genotype diversity in gastroenteritis outbreaks in Alberta, Canada: 2012–2018
Source: BMC Infect Dis. 2019 Feb 19;19:177. doi: 10.1186/s12879-019-3792-y (PMC6381812; doi:10.1186/s12879-019-3792-y)
Supplement: Supplementary file 3 — Figure S3. Description of data: Analysis of variable sites in capsid sequences of GII.P16/GII.4 Sydney recombinants compared to GII.Pe/GII.4 Sydney strains. Tables depict all variable amino acid residues identified in the alignment used to construct the tree shown in Additional file 2. Evolving amino acid positions previously reported by Lindesmith et al. [33] to play a role in the evasion of antibody immune responses are shown in red. (PDF 80 kb) [file 12879_2019_3792_MOESM3_ESM.pdf]

Name	Year	ORF1	6	9	11	15	23	33	54	106	119	126	145	174	185	208	242	291	293	294	297	304	309	310	315	317
MG214988.1 GII.Pe GII.4Syd/JN010/CN	2017	GII.Pe	S	N	S	A	N	V	N	Q	V	P	V	P	P	P	F	T	V	T	H	A	S	N	E	I
MH469206.1 GII.Pe GII.4Syd/GZ2017-L615/CN	2017	GII.Pe	S	N	S	A	N	V	N	Q	V	P	V	P	P	P	F	T	I	T	H	A	S	N	E	I
KX657729.1 GII.Pe GII.4Syd/16-CL-1/TW	2016	GII.Pe	S	N	S	A	N	V	N	Q	V	P	V	P	P	P	F	T	I	T	H	A	S	N	E	I
KX657737.1 GII.Pe GII.4Syd D031/TW	2016	GII.Pe	S	N	S	A	N	V	N	Q	V	P	V	P	P	P	F	T	I	T	H	A	S	N	E	I
KU678205.1 GII.Pe GII.4Syd/16-J-1/TW	2016	GII.Pe	S	N	S	A	N	V	N	Q	V	P	V	P	P	P	F	T	I	T	H	A	S	N	E	I
KX158279.1 GII.Pe GII.4Syd/13-38/CA	2015	GII.Pe	S	N	S	A	N	V	N	Q	V	P	V	P	P	P	F	T	I	T	R	A	N	N	E	I
LC066046.2 GII.Pe GII.4Syd/Osaka/OSF78/JP	2015	GII.Pe	S	N	S	A	N	V	N	Q	V	P	V	P	P	P	F	T	T	T	H	A	S	N	E	I
LC177659.1 GII.Pe GII.4Syd/NVN15.1296/VN	2015	GII.Pe	S	N	S	A	N	V	S	Q	V	P	V	P	P	T	F	T	I	T	R	A	N	N	E	I
MH469167.1 GII.Pe GII.4Syd/GZ2015-L400/CN	2015	GII.Pe	S	N	S	A	N	V	S	Q	V	P	V	P	P	P	F	T	I	T	R	A	S	N	E	I
KY488575.1 GII.Pe GII.4Syd/D001/TW	2014	GII.Pe	S	N	S	A	N	V	N	Q	V	P	V	P	P	P	F	T	I	T	R	A	S	N	E	I
KT202795.1 GII.Pe/GII.4Syd/GZ2014-L122/CN	2014	GII.Pe	S	N	S	A	N	V	N	Q	V	P	V	P	P	P	F	T	I	T	R	A	S	N	E	I
KT202798.1 GII.Pe GII.4Syd/GZ2014-L307/CN	2014	GII.Pe	S	N	S	A	N	V	N	Q	V	P	V	P	P	P	F	T	I	T	R	A	S	N	E	I
KX354033.1 GII.4Syd/2013-SP-0036 010913 HI/US	2013	GII.Pe	S	N	S	A	N	V	N	Q	I	P	I	S	P	P	F	T	I	T	R	A	N	N	E	I
KR131783.1 GII.4Syd/Pune-131742/IN	2013	GII.Pe	S	N	S	A	N	V	N	Q	I	P	I	S	P	P	F	T	I	T	R	A	N	N	E	I
KR904223.1 GII.4Syd/Johannesburg 12163/ZA	2013	GII.Pe	S	N	S	A	N	V	N	Q	I	P	I	S	P	P	F	T	I	T	R	A	N	N	E	I
JX459908.1 GII.Pe GII.4Syd/NSW0514/AU	2012	GII.Pe	S	N	S	A	N	V	N	Q	V	P	V	P	P	P	F	T	I	T	R	A	N	D	E	I
KF509946.3 GII.Pe GII.4Syd/AlbertaEI063/CA	2012	GII.Pe	S	N	S	A	N	V	N	Q	V	P	V	P	P	P	F	T	I	T	R	A	N	N	E	I
JX459907.1 GII.Pe GII.4Syd/Woonona/NSW3309/AU	2012	GII.Pe	S	N	S	A	N	V	N	Q	V	P	V	S	P	P	F	T	I	T	R	A	N	N	D	I
KF060080.1 GII.4Syd/NSW628G/AU	2012	GII.Pe	S	N	S	A	N	A	N	Q	V	P	V	S	P	P	F	T	I	T	R	A	N	N	E	I
KC517370.1 GII.4Syd/CGMH60/TW	2012	GII.Pe	S	N	S	A	N	V	N	Q	V	S	I	S	L	P	F	T	I	T	R	A	N	N	E	I
KF060062.1 GII.4Syd/NSW558V/AU	2012	GII.Pe	S	N	T	A	N	V	N	Q	I	P	I	S	P	P	F	T	I	T	R	A	N	N	E	T
KF060126.1 GII.4Syd/NLV-12-1056/NZ	2012	GII.Pe	S	N	S	A	N	V	N	E	I	P	I	S	P	P	F	T	I	T	R	A	N	N	E	I
KX354017.1 GII.4Syd/2012-SP-0566 103112 TX/US	2012	GII.Pe	S	N	S	A	N	V	N	Q	I	P	I	S	P	P	F	T	I	T	R	A	N	N	E	I
AB972499.1 GII.Pe GII.4Syd/HK01/JP	2011	GII.Pe	S	N	S	A	N	V	N	Q	V	P	I	S	P	P	F	T	I	T	R	A	N	N	E	I
KX354140.1 GII.4Syd2015-SP-0399 122815 OR/US	2015	GII.P16	S	S	S	A	N	V	N	Q	I	P	I	S	P	P	F	T	I	T	R	A	N	N	E	I
KX354146.1 GII.4Syd/2015-SP-0385 111315 LAC/US	2015	GII.P16	S	N	S	A	N	V	N	Q	I	P	I	P	P	P	L	T	I	T	R	A	N	N	E	I
KY947550.1 GII.P16 GII.4Syd/Pasadena 3477/US	2015	GII.P16	S	N	S	A	N	V	N	Q	I	P	I	P	P	P	F	T	I	T	R	A	N	N	E	I
KY887601.1 GII.P16 GII.4Syd/NOR-2565/UK	2016	GII.P16	S	N	S	A	N	V	N	Q	I	P	I	S	P	P	F	T	I	T	R	A	N	N	E	I
LC153121.1 GII.P16 GII.4Syd/OH16002/JP	2016	GII.P16	S	N	S	A	N	V	N	Q	I	P	I	S	P	P	F	I	I	T	R	A	N	N	E	I
LC153122.1 GII.P16 GII.4Syd/OC16023/JP	2016	GII.P16	S	N	S	A	N	V	N	Q	I	P	I	S	P	P	F	T	I	T	R	V	N	N	E	I
LC175468.1 GII.P16 GII.4Syd/Kawasaki194/JP	2016	GII.P16	S	N	S	A	N	V	N	Q	I	P	I	S	P	P	F	T	I	T	R	A	N	N	E	I
AlbertaEI084/CA	2016	GII.P16	N	N	S	A	N	V	N	Q	I	P	I	S	P	P	F	T	I	T	R	A	N	N	E	I
AlbertaEI277/CA	2016	GII.P16	S	N	S	A	N	V	N	Q	I	P	I	S	P	P	F	T	I	T	R	A	N	N	E	I
LC331997.1 GII.P16 GII.4Syd/AichiF25/JP	2017	GII.P16	S	N	S	A	N	V	N	Q	I	P	I	S	P	P	F	T	I	T	R	A	N	N	E	I
MG002630.1 GII.P16 GII.4Syd/BNE1/AU	2017	GII.P16	S	N	S	A	N	V	N	Q	I	P	I	S	P	P	F	T	I	T	R	A	N	N	E	I
MG002633.1 GII.P16 GII.4Syd/BNE4/AU	2017	GII.P16	S	N	S	A	N	V	N	Q	I	P	I	S	P	P	F	T	I	T	H	A	N	N	E	I
AlbertaEI487/CA	2017	GII.P16	S	N	S	A	S	V	N	Q	I	P	I	S	P	P	F	T	I	T	R	A	N	N	E	I
AlbertaEI231/CA	2018	GII.P16	S	N	S	A	N	V	S	Q	I	P	I	S	P	P	F	T	I	T	H	A	N	N	E	I
GU445325.2 GII.P4NO GII.4NO/New Orleans1805/US	2009	GII.P4 New Orleans	S	N	S	T	N	V	N	Q	I	P	I	S	P	P	F	T	I	P	R	A	N	S	E	I

Name	Year	ORF1	322	330	333	341	350	351	359	368	372	373	377	380	393	396	404	412	413	414	436	451	512	534	539	540
MG214988.1 GII.Pe GII.4Syd/JN010/CN	2017	GII.Pe	G	I	M	D	T	V	A	E	N	N	A	N	G	H	V	N	T	P	S	C	N	A	A	L
MH469206.1 GII.Pe GII.4Syd/GZ2017-L615/CN	2017	GII.Pe	G	I	M	D	T	V	A	E	N	N	A	N	G	H	V	N	T	P	S	C	N	A	A	L
KX657729.1 GII.Pe GII.4Syd/16-CL-1/TW	2016	GII.Pe	G	I	M	D	T	V	A	E	N	N	A	N	G	H	V	N	T	P	S	C	N	A	A	L
KX657737.1 GII.Pe GII.4Syd D031/TW	2016	GII.Pe	G	I	M	D	T	V	A	E	N	N	A	N	G	H	V	N	T	P	S	C	N	A	A	L
KU678205.1 GII.Pe GII.4Syd/16-J-1/TW	2016	GII.Pe	G	V	V	D	T	V	A	E	N	H	A	N	G	H	V	N	T	P	S	C	N	T	A	L
KX158279.1 GII.Pe GII.4Syd/13-38/CA	2015	GII.Pe	G	I	V	D	T	V	A	E	D	H	A	N	S	H	V	N	T	P	S	C	N	T	A	L
LC066046.2 GII.Pe GII.4Syd/Osaka/OSF78/JP	2015	GII.Pe	G	I	V	D	T	V	A	E	N	H	A	N	G	H	V	N	T	P	S	C	N	T	A	L
LC177659.1 GII.Pe GII.4Syd/NVN15.1296/VN	2015	GII.Pe	G	I	M	D	T	V	A	E	D	H	A	N	S	H	V	N	T	P	S	C	N	T	A	L
MH469167.1 GII.Pe GII.4Syd/GZ2015-L400/CN	2015	GII.Pe	G	I	M	D	T	V	A	E	D	H	A	N	S	H	V	N	T	P	S	C	N	T	A	L
KY488575.1 GII.Pe GII.4Syd/D001/TW	2014	GII.Pe	G	I	M	D	T	V	A	E	D	H	A	N	S	H	V	N	T	P	S	C	N	T	A	L
KT202795.1 GII.Pe/GII.4Syd/GZ2014-L122/CN	2014	GII.Pe	G	I	V	D	T	V	A	E	D	R	A	N	G	H	V	N	I	P	S	C	N	T	A	L
KT202798.1 GII.Pe GII.4Syd/GZ2014-L307/CN	2014	GII.Pe	G	I	V	D	T	V	A	E	D	H	A	N	G	H	V	N	T	P	S	C	N	T	A	L
KX354033.1 GII.4Syd/2013-SP-0036 010913 HI/US	2013	GII.Pe	G	I	M	D	T	V	A	E	D	H	A	N	S	H	V	N	T	H	S	C	N	T	V	V
KR131783.1 GII.4Syd/Pune-131742/IN	2013	GII.Pe	G	I	M	D	T	V	A	E	D	H	A	N	S	H	V	N	T	H	S	C	N	T	V	V
KR904223.1 GII.4Syd/Johannesburg 12163/ZA	2013	GII.Pe	G	I	M	D	T	V	A	E	D	H	A	N	S	H	V	N	T	H	S	C	Y	T	A	L
JX459908.1 GII.Pe GII.4Syd/NSW0514/AU	2012	GII.Pe	G	I	V	D	T	V	A	E	D	R	A	N	G	H	V	N	T	H	S	C	N	T	A	V
KF509946.3 GII.Pe GII.4Syd/AlbertaEI063/CA	2012	GII.Pe	G	I	V	D	T	V	A	E	D	H	A	N	G	H	V	N	T	H	S	C	N	T	A	V
JX459907.1 GII.Pe GII.4Syd/Woonona/NSW3309/AU	2012	GII.Pe	G	I	M	D	T	V	A	E	D	H	A	N	S	H	V	N	T	H	S	C	N	T	V	V
KF060080.1 GII.4Syd/NSW628G/AU	2012	GII.Pe	G	I	M	D	T	V	A	E	D	H	A	N	S	H	V	N	T	H	S	C	N	T	V	V
KC517370.1 GII.4Syd/CGMH60/TW	2012	GII.Pe	G	I	M	D	T	M	A	E	D	H	A	N	G	H	V	N	T	H	P	R	N	T	V	V
KF060062.1 GII.4Syd/NSW558V/AU	2012	GII.Pe	G	I	M	D	T	V	A	E	D	H	A	N	S	H	V	N	T	H	S	C	N	T	V	V
KF060126.1 GII.4Syd/NLV-12-1056/NZ	2012	GII.Pe	G	I	M	D	T	V	A	E	D	H	A	N	S	H	V	N	T	H	S	C	N	T	V	V
KX354017.1 GII.4Syd/2012-SP-0566 103112 TX/US	2012	GII.Pe	G	I	M	D	T	V	A	E	D	H	A	N	S	H	V	N	T	H	S	C	N	T	V	V
AB972499.1 GII.Pe GII.4Syd/HK01/JP	2011	GII.Pe	G	I	M	D	T	V	A	E	D	H	A	N	S	H	V	N	T	H	S	C	N	T	V	V
KX354140.1 GII.4Syd2015-SP-0399 122815 OR/US	2015	GII.P16	A	I	M	D	T	V	A	E	D	H	A	N	S	H	V	N	T	H	S	C	N	T	V	V
KX354146.1 GII.4Syd/2015-SP-0385 111315 LAC/US	2015	GII.P16	G	I	M	D	T	V	A	E	D	H	A	N	S	H	V	N	T	H	S	C	N	T	A	V
KY947550.1 GII.P16 GII.4Syd/Pasadena 3477/US	2015	GII.P16	G	I	M	D	I	V	A	E	D	H	A	N	S	H	V	N	T	H	S	C	N	T	A	V
KY887601.1 GII.P16 GII.4Syd/NOR-2565/UK	2016	GII.P16	G	I	M	D	T	V	A	E	D	H	A	N	S	H	V	N	T	H	S	C	N	T	V	V
LC153121.1 GII.P16 GII.4Syd/OH16002/JP	2016	GII.P16	G	I	M	D	T	V	A	E	D	H	A	N	S	H	V	N	T	H	S	C	N	T	V	V
LC153122.1 GII.P16 GII.4Syd/OC16023/JP	2016	GII.P16	G	I	M	D	T	V	A	E	D	H	A	N	S	H	V	N	T	H	S	C	N	T	A	V
LC175468.1 GII.P16 GII.4Syd/Kawasaki194/JP	2016	GII.P16	G	I	M	D	T	V	A	E	D	H	A	N	S	H	V	N	T	H	S	C	N	T	A	V
AlbertaEI084/CA	2016	GII.P16	G	I	M	D	T	V	A	E	D	H	A	N	S	H	V	N	T	H	S	C	N	T	A	V
AlbertaEI277/CA	2016	GII.P16	G	I	M	D	T	V	A	E	D	H	A	D	S	H	A	N	T	H	S	C	N	T	A	V
LC331997.1 GII.P16 GII.4Syd/AichiF25/JP	2017	GII.P16	G	I	M	D	T	V	A	E	D	H	A	N	S	H	V	N	T	H	S	C	N	T	V	V
MG002630.1 GII.P16 GII.4Syd/BNE1/AU	2017	GII.P16	G	I	M	D	T	V	A	E	D	H	A	N	S	H	V	N	T	H	S	C	N	T	V	V
MG002633.1 GII.P16 GII.4Syd/BNE4/AU	2017	GII.P16	G	I	M	D	T	V	A	E	N	H	A	N	S	H	V	N	T	H	S	C	N	T	V	V
AlbertaEI487/CA	2017	GII.P16	G	I	V	D	T	V	A	E	D	H	A	N	S	H	V	N	T	H	S	C	N	T	V	V
AlbertaEI231/CA	2018	GII.P16	G	I	M	D	T	V	A	E	N	H	A	N	S	H	V	S	T	H	S	C	N	T	V	V
GU445325.2 GII.P4NO GII.4NO/New Orleans1805/US	2009	GII.P4 New Orleans	G	I	V	N	T	V	S	A	D	N	T	N	S	P	V	N	I	H	S	C	N	T	A	L

Comparison of variable sites among GII.4 Sydney capsid sequences collected in different countries and different years. Evolving positions previously reported by Lindesmith *et al.*(1) to play a role in the evasion of antibody immune responses are shown in red.

(1) Lindesmith LC, Brewer-Jensen PD, Mallory ML, Debbink K, Swann EW, Vinjé J, Baric RS. J Infect Dis. 2018 Mar 13;217(7):1145-1152.
